# Novel Additives in Copper Electrorefining—Small Laboratory Scale

**DOI:** 10.3390/ma17061262

**Published:** 2024-03-08

**Authors:** Patrycja Kowalik, Dorota Kopyto, Katarzyna Leszczyńska-Sejda, Wojciech Simka

**Affiliations:** 1 Łukasiewicz Research Network—Institute of Non-Ferrous Metals, Sowińskiego 5, 44-100 Gliwice, Poland; dorota.kopyto@imn.lukasiewicz.gov.pl (D.K.); katarzyna.leszczynska-sejda@imn.lukasiewicz.gov.pl (K.L.-S.); 2 Department of Inorganic Chemistry, Analytical Chemistry and Electrochemistry, Faculty of Chemistry, Silesian University of Technology, B. Krzywoustego 6, 44-100 Gliwice, Poland; wojciech.simka@polsl.pl

**Keywords:** copper, electrorefining, electrodeposition, electrolyte additive, addition agent, ionic liquid

## Abstract

This research aimed to evaluate the effectiveness of new organic substances, including a novel ionic liquid based on polyhexamethylenebiguanidine, polyhexamethyleneguanidine, and safranin in the copper electrorefining process. Experiments were conducted on a small laboratory scale using industrial copper anodes. Single doses of new additives did not improve process indicators (current efficiency, average cell voltage, specific energy consumption) or the quality of copper cathode deposits. However, a combination of a new ionic liquid based on polyhexamethylenebiguanidine and thiourea resulted in a satisfactory current efficiency of 97%, an average cell voltage of 0.110 V, a low specific energy consumption index of approximately 100 kWh/t_Cu_, and smooth cathode surfaces. These results were superior to those obtained with industrial additives (bone glue and thiourea). The findings enhance our understanding of how these substances influence the electrorefining process and suggest the potential for more efficient and sustainable methods. Further research is recommended to validate these findings and explore their industrial applications.

## 1. Introduction

Electrorefining consists of the electrolytical dissolving of copper from an impure copper anode into an electrolyte. The dissolved copper is then selectively electrodeposited on the cathode. The electrorefining process is the final step of copper purification from accompanying impurities. They contaminate the produced copper cathodes and negatively affect their physical, chemical, and mechanical properties. The technical and economic indicators of the electrorefining process, as well as the quality of the electrolytic copper cathodes, depend on various factors, one of which is the type and activity of organic compounds added to the electrolyte [1,2]. The addition agents are introduced to the electrolyte to improve the external appearance of the cathodes. These additives adsorb on the cathode surface and take part in the electrochemical crystallization process. They work toward preventing the formation of dendrites on the cathode deposit and affect its crystalline structure [3,4,5]. During the electrorefining process, copper is deposited on the cathode in the form of crystals, which tend to grow into irregular shapes in the form of balls, cones, and thick growths, known as dendrites. This phenomenon is undesirable due to the fact that these forms have the ability to capture anode slime, which can be present in the form of a suspension in the electrolyte. In addition, dendrites are the main cause of short circuits between the cathode and anode [5,6,7]. The electrolyte additives enable the deposition of fine-grained copper cathodes and a smooth surface free from dendrites, and they counteract the growth of dendrites. They can be divided into leveling agents, ensuring the uniformity of the cathode deposit, and grain-refining agents, affecting the orientation of the crystals [8,9,10]. Currently known inhibitors of the industrial copper electrorefining process include glue, thiourea, avitone, and also inorganic chloride ions [11,12,13,14,15,16,17,18]. The main purpose of adding chloride ions is to precipitate silver by forming silver chloride, which settles at the bottom of the electrolytic cell. Additionally, chloride ions improve and brighten cathode deposits when combined with organic additives [19]. Researchers around the world are continuously seeking innovative organic substances that can act as inhibitors in the electrorefining process. A wide range of studies are being conducted on these substances, including groups of compounds such as ionic liquids [20,21,22,23] and deep eutectic solvents [24,25,26,27,28,29,30], as well as individual compounds, including polyethylene glycol (PEG) [31,32,33], lignin-based biopolymer (DP 2782) [34], bis(3-sulfopropyl) disulfide Na_2_[SO_3_(CH_2_)_3_S]_2_ (SPS) [35], 3-N,N-dimethylaminodithiocarbamoyl-1-propanesulphonic acid (DPS) [32], (NaO_3_S-(CH_2_)_3_-S-(CH_2_)_3_-SO_3_Na) 3,3-thiobis-1-propanesulfonic acid (TBPS) [36], and the mixture of triethyl-benzyl-ammonium chloride (TEBA) with hydroxyethylated-2-butyne-l,4-diol “Ferasine” (IT-85) [37]. However, the authors’ current research stands out as it involves the use of a novel ionic liquid based on polyhexamethylenebiguanidine [38]. This innovative approach is aimed at replacing or reducing the doses of additives that are currently used in global copper electrorefineries, which are known to have numerous disadvantages and inconveniences. This research could potentially lead to significant advancements in the field of copper electrorefining.

Therefore, the main aim of the research work undertaken was to test new organic substances: an ionic liquid based on polyhexamethylenebiguanidine [38], polyhexamethyleneguanidine, safranin, and their combinations with other inhibitors such as bone glue and thiourea as addition agents in copper electrorefining. The tests were conducted on a small laboratory scale and used industrial copper anodes from domestic copper electrorefineries. The experiments showed an improvement in current efficiency and the morphology of the obtained copper cathode deposits as well as a reduction in average cell voltage and specific energy consumption. All the obtained results, both process indicators and the appearance of cathodes, were compared with those obtained in trials using a classic set of additives used in the industry—bone glue and thiourea.

## 2. Materials and Methods

**Figure 1:**
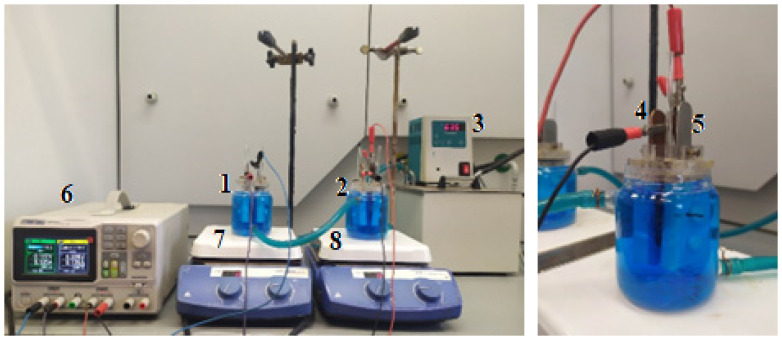
A station for testing the processes of copper electrorefining on a small laboratory scale: (1) and (2) glass electrolyzers, (3) ultra-thermostat, (4) anode, (5) cathode, (6) power supply, (7) and (8) magnetic stirrers.

The apparatus used in the copper electrorefining trials is presented in Figure 1. A station was equipped with two glass electrolyzers (1, 2), each with an active volume of 180 cm^3^, fitted with a heating jacket powered by an ultra-thermostat (3).

In the studies, the following electrodes were used: an anode (4) made of industrial anode copper and a cathode (5) made of copper foil about 0.2 mm thick. The composition of industrial anode copper was: 0.12% Pb; 0.13% As; 0.17% Ni; 0.015% Sb and 0.09% O. The electrodes were installed in PTFE holders, which restricted their active surface area to approximately 5 cm^2^. They were attached to the covers of the electrolyzers in a way that ensured a constant distance between the anode and cathode surfaces—2.5 cm. The electrode system was powered by a direct current from the SPD3303X power supply (Siglent, Waszawa, Poland) (6). Before starting the electrorefining process, the cathode was degreased with acetone (Stanlab, Lublin, Poland). The electrolyte, used in the studies, was prepared from the following reagents: copper sulfate (Chempur, Piekary Ślaskie, Poland), sulfuric acid (Pol-Aura, Zabrze, Poland), and distilled water. Its composition was determined as the following: Cu^2+^—44.5 g/dm^3^ and H_2_SO_4_—157.3 g/dm^3^. The solution in the electrolyzers was mixed with magnetic stirrers (7, 8).

The technological conditions of all conducted copper electrorefining trials were as follows: electrolyte temperature 60 °C, current density 250 A/m^2^, current intensity 0.125 A, and electrorefining time 5 h. The electrodes were weighed before and after each trial to determine the cathodic and anodic efficiency. The smoothness of the cathode deposits obtained in individual trials was examined using a Hommel Tester—2000 profilograph (Hommel Werke GmbH, Villingen-Schwenningen, Germany), determining the following profile roughness parameters: R_A_ [µm]—the arithmetic average of profile height deviations from the mean line—R_Z_ [µm]—the average distance of the five highest peaks on the surface, from the five lowest points of the recesses on the measurement section—and Rm [µm]—the distance between two parallel lines, one of which passes through the highest peak, with the other passing through the lowest point of the recesses. Measurements were performed with the movement of the measuring tip across the sample surface. The length of the single measurement section was 0.8 mm. Measurements were carried out in the upper, middle and lower zone of the examined cathode deposit, with 6 measurements being performed for each sample and the tester sensor shift being maintained horizontally and vertically in relation to the electrode position in the electrolyzer. The result was given as the arithmetic mean of the measurements made. Due to the limited measurement range of the profilograph, the measurement of surface parameters of some of the obtained cathodes characterized by a large surface irregularity was impossible. For this reason, an additional point assessment of the quality of the cathode deposit was introduced. In this method, subjectively, the appearance of the cathode surface (Table 1) and the crystalline structure (Table 2) were assessed according to the following adopted point scale:

The organic substances used in the studies were an ionic liquid based on polyhexamethylene-biguanidine (98.8%), polyhexamethyleneguanidine (50% aqueous solution), and safranin. These substances were provided by the Łukasiewicz–Łódź Institute of Technology. Additionally, bone glue (Kremer, Kraków, Poland) and thiourea (Warchem, Zakręt, Poland), which are inhibitors commonly used in electrorefineries around the world, were also used in the research as inhibitors. All organic substances were introduced into the electrolyte at different concentrations, individually and in sets, also in combination with bone glue and thiourea. Their initial concentrations and the sets of additives that were introduced into the copper electrorefining process are presented in Table 3. The symbols assigned to them are as follows: IL—ionic liquid based on polyhexamethylenebiguanidine; P—polyhexamethyleneguanidine; S—safranin; BG—bone glue; and T—thiourea. The method of serial dilutions was employed to prepare inhibitor solutions with such low concentrations.

The appearance of all obtained cathode deposits is shown in the photos. In each electrorefining trial, the impact of new sets of additives on the appearance and crystalline structure of copper and on process indicators, i.e., current efficiency *η* (%), specific energy consumption SEC (kWh/t_Cu_), and average cell voltage *V_cell_* (*V*), was examined.

The values of current efficiency *η* (%) were calculated with the following equation:

(1) $$\eta =\frac{m_{p}}{z\;\cdot \;I\;\cdot \;t}\cdot 100\%,$$

where *m_p_* is the quantity of copper deposited at the cathode, *z* is the electrochemical equivalent of the copper which is determined using Faraday’s law, *I* is the amperage, and *t* is the process time.

The values of specific energy consumption SEC (kWh/t_Cu_) were calculated with the following equation:

(2) $$\mathrm{SEC}=\frac{V_{cell}}{\eta \;\cdot z}$$

where *V_cell_* is the average cell voltage observed during the process, and *η* is the current efficiency.

All results gathered, encompassing both process indicators and the visual assessment of cathodes, were juxtaposed with the outcomes from experiments that utilized a traditional set of additives prevalent in the industry, bone glue and thiourea.

## 3. Results and Discussion

### 3.1. Electrolysis without and with a Classic Set of Additives

**Figure 2:**
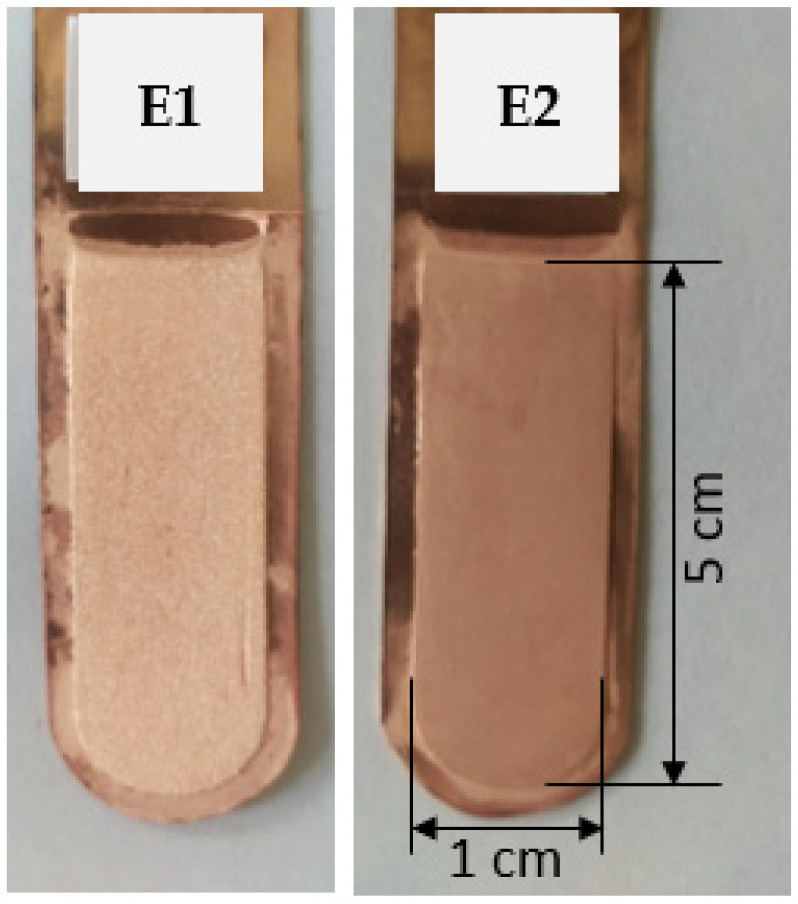
Appearance of the obtained cathode Cu deposits: (E1) without organic additives and (E2) with the addition of bone glue and thiourea.

Two electrorefining trials were initially conducted: the first one utilized a synthetic electrolyte without organic additives (E1), while the second one incorporated bone glue and thiourea (E2). The appearance of the copper cathodes is presented in Figure 2. The height of each electrodeposited copper deposit was 5 cm, and the width was 1 cm, as shown in the photo from the E2 trial. Table 4 presents the average profile roughness parameters of the obtained copper deposits.

Measurements of average profile roughness parameters and visual evaluations of copper cathode deposits indicated that incorporating bone glue and thiourea enhances the aesthetics of Cu deposits, resulting in a fine-grained, smooth, and matte finish. Without organic additives, individual irregularities formed on the shiny cathode surface, where noticeable crystals could be seen.

Table 5 presents the process indicators for the E1 and E2 electrorefining processes, as well as the evaluation of the cathode deposit quality.

Upon analysis of the average cell voltage values during the E2 test, it was evident that the inclusion of bone glue and thiourea in this trial contributed to a reduction in voltage. Conversely, the electrorefining process without additives (E1) was characterized by a higher average cell voltage and an increase in specific energy consumption.

### 3.2. Electrolysis with a Single Addition of the Inhibitor

The experiments were conducted with a single addition of a new electrolyte additive to each trial. Safranin, polyhexamethyleneguanidine, and an ionic liquid based on polyhexamethylenebiguanidine were used in the studies.

#### 3.2.1. Safranin

**Figure 3:**
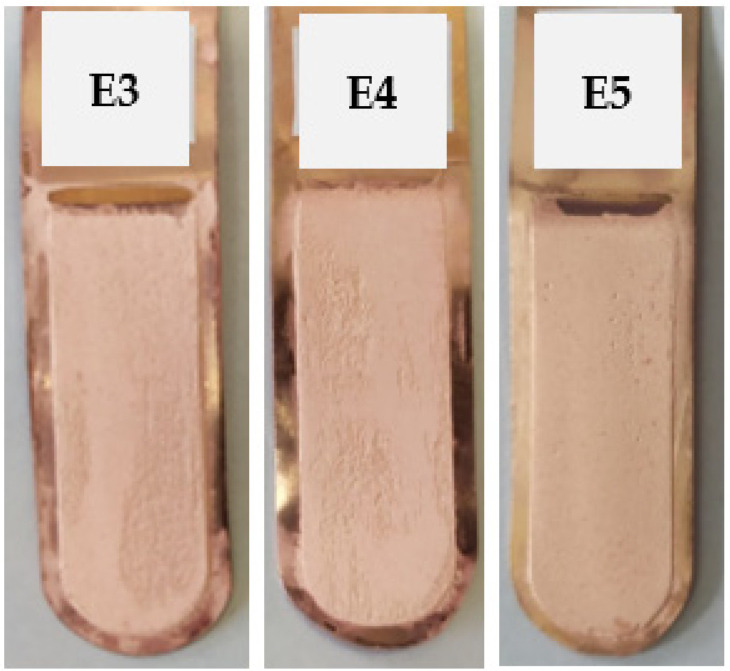
Appearance of the obtained cathode Cu deposits with the addition of safranin at concentrations of 10 mg/dm^3^ (E3), 25 mg/dm^3^ (E4), and 50 mg/dm^3^ (E5).

Electrorefining trials E3, E4, and E5 were conducted with the addition of safranin at the concentrations listed in Table 3. The appearance of the cathode copper deposits is presented in Figure 3. Table 6 presents the average profile roughness parameters of the obtained electrolytic copper deposit.

The increase in the initial concentration of safranin in the electrolyte improved the appearance and form of the obtained cathode copper. The addition of 50 mg/dm^3^ of safranin resulted in the obtained copper being characterized by a smooth surface with noticeable, small, and single scratches. However, the surface of the copper in the E3 and E4 trials had unevenly applied sediment on its entire surface, thus resulting in numerous irregularities. Table 7 presents the process indicators for the E3, E4, and E5 electrorefining processes, as well as the evaluation of cathode deposit quality.

The *V_cell_* values declined as the initial concentration of safranin in the electrolyte increased. In addition, the SEC also decreased with an increase in the initial concentration of this inhibitor. The current efficiencies in trials E3–E5 exceeded 98%.

#### 3.2.2. Polyhexamethyleneguanidine

**Figure 4:**
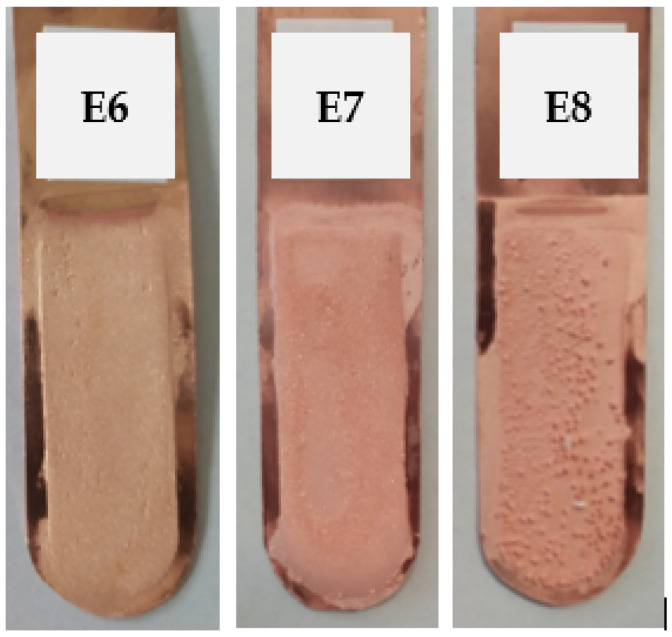
Appearance of the obtained cathode Cu deposits with the addition of polyhexamethyleneguanidine at concentrations of 0.05 mg/dm^3^ (E6), 0.25 mg/dm^3^ (E7), and 0.50 mg/dm^3^ (E8).

Electrorefining experiments E6, E7, and E8 were carried out with the addition of polyhexamethyleneguanidine (P) at the concentrations specified in Table 3. The visual representation of the cathode copper deposits can be seen in Figure 4. The average profile roughness parameters of the resulting electrolytic copper deposit are shown in Table 8.

An increase in the concentration of polyhexamethyleneguanidine in the electrolyte led to a decline in the appearance and form of the resulting cathode copper. The copper produced with the addition of 0.05 mg/dm^3^ P was distinguished by a smooth surface with small, isolated scratches. The addition of 0.25 mg/dm^3^ P caused growths to form on the edges and uneven copper deposition across the entire surface. However, the introduction of 0.50 mg/dm^3^ P resulted in bubble formation across the entire cathode surface. Due to the numerous bubbles and irregularities formed in E8, it was impossible to measure the roughness of this cathode surface. In the case of the deposit from E7, a horizontal profile roughness measurement could not be conducted. Table 9 presents the process indicators for the E6, E7, and E8 electrorefining processes, as well as the evaluation of the Cu deposit quality.

The analysis of the *V_cell_* values in all three trials, with the addition of polyhexamethyleneguanidine to the electrolyte, showed a distinct increase in *V_cell_*. This increase corresponded with the rise in the initial concentration of the inhibitor in the electrolyte. The increase in the concentration of the P in the electrolyte exerted a polarizing effect on the cathode process. In a similar vein, the SEC also increased. The *η* in trials E6–E8 exceeded 97%.

#### 3.2.3. Ionic Liquid Based on Polyhexamethylenebiguanidine

**Figure 5:**
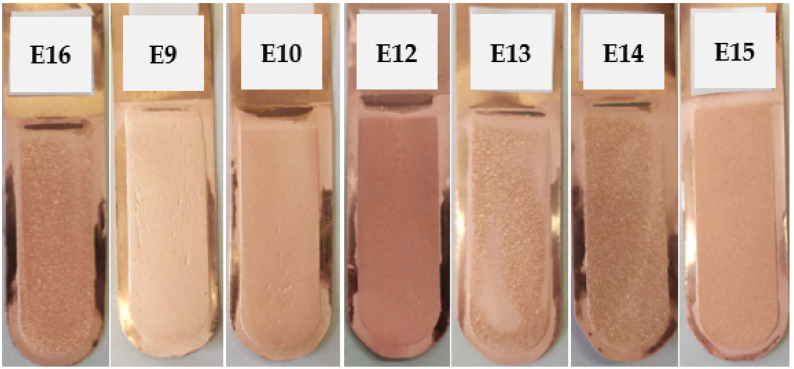
Appearance of the obtained cathode Cu deposits with the addition of ionic liquid based on polyhexamethylenebiguanidine at concentrations of 0.005 mg/dm^3^ (E16), 0.01 mg/dm^3^ (E9), 0.05 mg/dm^3^ (E10), 0.50 mg/dm^3^ (E12), 5.0 mg/dm^3^ (E13), 50.0 mg/dm^3^ (E14), and 250.0 mg/dm^3^ (E15).

Electrorefining trials were performed using an ionic liquid based on polyhexamethylenebiguanidine at the concentrations outlined in Table 3. The appearance of the cathode copper deposits is illustrated in Figure 5. The average profile roughness parameters of the obtained electrolytic copper deposits are detailed in Table 10.

Measurements of the average profile roughness indicated that the smoothest surfaces of the cathode deposits were obtained in tests E9 and E10. The deposit from test E13 exhibited the roughest surface. The introduction of a new inhibitor in a quantity exceeding 5.0 mg/dm^3^ led to a brightening of the deposits.

Table 11 presents the process indicators for the E16, E9, E10, E12, E13, E14, and E15 electrorefining processes, as well as the evaluation of cathode deposit quality.

The lowest *V_cell_* value was recorded in test E16, where the initial concentration of the tested ionic liquid was at its minimum—0.005 mg/dm^3^. The average cell voltages in tests E9, E10, E12, E13, and E14 ranged from 0.142 to 0.171 V, with values of SEC between 122.6 and 148.8 kWh/t_Cu_. Adding the highest concentration of the IL—250 mg/dm^3^—to the electrolyte resulted in an increase in *V_cell_* to a value of 0.238 V. Current efficiencies were consistently above 97%, with the sole exception being test E15, where this value was 94.1%.

Single doses of inhibitors (safranin, polyhexamethyleneguanidine, and an ionic liquid based on polyhexamethylenebiguanidine) did not improve the process indicators. The appearance of obtained cathodes, depending on the initial concentration of the selected inhibitor, was characterized by minor defects, scratches, unevenness, or larger bubbles and dendrites. Therefore, further trials were conducted, introducing sets of two inhibitors into the electrolyte.

### 3.3. Electrolysis with the Addition of Two Inhibitors

Sets of new inhibitors with different concentrations were introduced into the electrolyte in the following configuration: ionic liquid based on polyhexamethylenebiguanidine and bone glue (IL + BG), ionic liquid based on polyhexamethylenebiguanidine and thiourea (IL + T), ionic liquid based on polyhexamethylenebiguanidine and safranin (IL + S), and ionic liquid based on polyhexamethylenebiguanidine and polyhexamethyleneguanidine (IL + P).

#### 3.3.1. Ionic Liquid Based on Polyhexamethylenebiguanidine and Bone Glue

**Figure 6:**
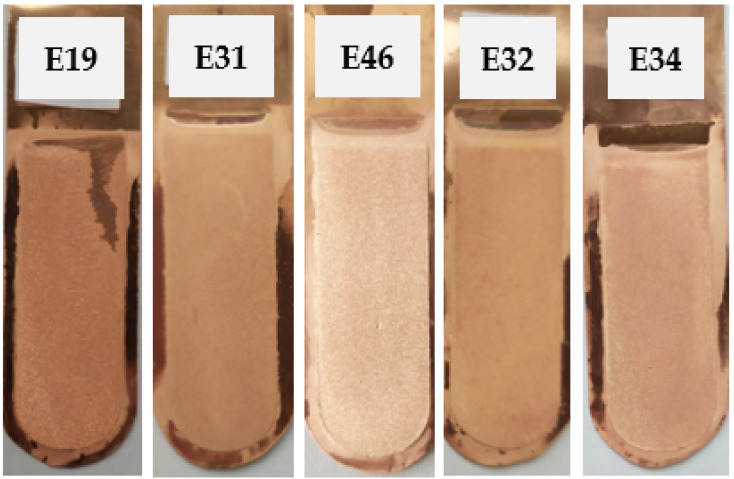
Appearance of the obtained cathode Cu deposits with the addition of ionic liquid based on polyhexamethylenebiguanidine (IL) and bone glue (BG) at concentrations of 0.005 mg/dm^3^ IL + 5.0 mg/dm^3^ BG (E19), 0.05 mg/dm^3^ IL + 5.0 mg/dm^3^ BG (E31), 0.15 mg/dm^3^ IL + 5.0 mg/dm^3^ BG (E46), 0.5 mg/dm^3^ IL + 5.0 mg/dm^3^ BG (E32), and 5.0 mg/dm^3^ IL + 5.0 mg/dm^3^ BG (E34).

The first set of inhibitors introduced into the electrolyte was a set consisting of an ionic liquid based on polyhexamethylenebiguanidine (IL) and bone glue (BG) at concentrations specified in Table 3. The appearance of the cathode copper deposits is presented in Figure 6. Table 12 presents the average profile roughness parameters of the obtained electrolytic copper deposit.

The obtained cathode deposits were characterized by a smooth structure, devoid of irregularities. The obtained copper cathodes were shiny. Deposits E31 and E32 exhibited the lowest values for the average profile roughness parameters of the obtained Cu deposits.

Table 13 presents the process indicators for the E19, E31, E46, E32, and E34 electrorefining processes, as well as the evaluation of the cathode deposit quality.

The *V_cell_* values consistently stayed within the range of 0.158–0.179 V. However, for E34, which utilized the highest concentration of ionic liquid in combination with bone glue, the value increased to 0.199 V. Notably, the *η* values were impressively high >98%.

#### 3.3.2. Ionic Liquid Based on Polyhexamethylenebiguanidine and Thiourea

**Figure 7:**
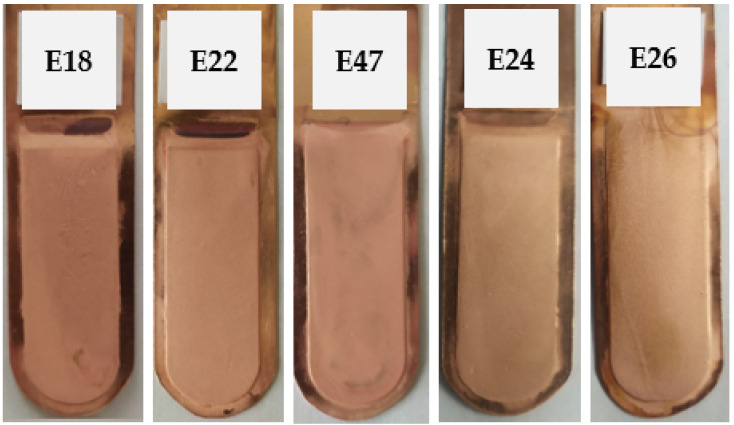
Appearance of the obtained cathode Cu deposits with the addition of ionic liquid based on polyhexamethylenebiguanidine (IL) and thiourea (T) at concentrations of 0.005 mg/dm^3^ IL + 5.0 mg/dm^3^ T (E18), 0.05 mg/dm^3^ IL + 5.0 mg/dm^3^ T (E22), 0.15 mg/dm^3^ IL + 5.0 mg/dm^3^ T (E47), 0.5 mg/dm^3^ IL + 5.0 mg/dm^3^ T (E24), and 5.0 mg/dm^3^ IL + 5.0 mg/dm^3^ T (E26).

The next set of inhibitors introduced into the electrolyte was a set consisting of an ionic liquid based on polyhexamethylenebiguanidine (IL) and thiourea (T) at the concentrations outlined in Table 3. The appearance of the cathode copper deposits is presented in Figure 7. Table 14 presents the average profile roughness parameters of the obtained electrolytic Cu deposit.

All deposits exhibited a smooth surface, free from defects and visible cracks. The average profile roughness parameters demonstrated low values, proving as beneficial as those observed in trials with classic inhibitors of the electrorefining process, such as bone glue and thiourea.

Table 15 presents the process indicators for the E18, E22, E47, E24, and E26 electrorefining processes, as well as the evaluation of the cathode deposit quality.

Satisfactory current indicators of the copper electrorefining process were obtained, both in the values of average cell voltage and specific energy consumption. These values were lower than those obtained in the E2 test with classic inhibitors of the electrorefining process.

#### 3.3.3. Ionic Liquid Based on Polyhexamethylenebiguanidine and Safranin

**Figure 8:**
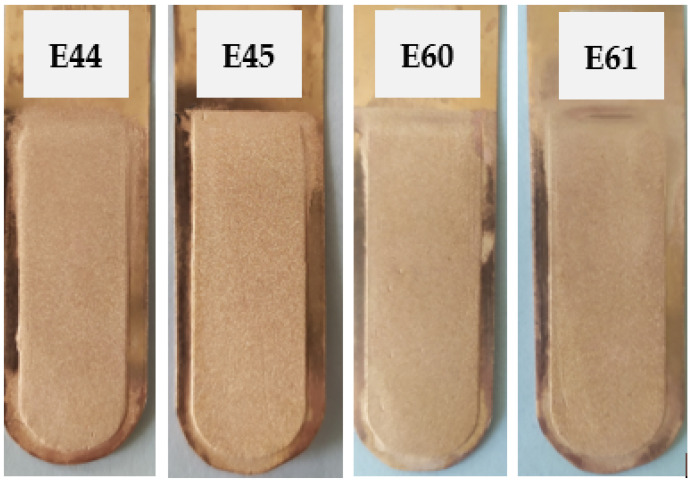
Appearance of the obtained cathode Cu deposits with the addition of ionic liquid based on polyhexamethylenebiguanidine (IL) and safranin (S) at concentrations of 0.005 mg/dm^3^ IL + 50 mg/dm^3^ S (E44), 0.05 mg/dm^3^ IL + 50 mg/dm^3^ S (E45), 0.15 mg/dm^3^ IL + 50 mg/dm^3^ S (E60), and 0.5 mg/dm^3^ IL + 50 mg/dm^3^ S (E61).

The next set of inhibitors introduced into the electrolyte was a set consisting of an ionic liquid based on polyhexamethylenebiguanidine (IL) and safranin (S) at the concentrations listed in Table 3. The appearance of the cathode copper deposits is presented in Figure 8. Table 16 presents the average profile roughness parameters of the obtained electrolytic copper deposit.

Deposits as equally smooth as those in the case of the E2 test with the classic addition of inhibitors were be obtained. Table 17 presents the process indicators for the E44, E45, E60, and E61 electrorefining processes, as well as the evaluation of the cathode deposit quality.

As the initial concentration of the ionic liquid increased in the presence of safranin, both the specific energy consumption and average cell voltage rose, from 0.108 to 0.201 V and from 93.1 to 172.1 kWh/t_Cu_, respectively.

#### 3.3.4. Ionic Liquid Based on Polyhexamethylenebiguanidine and Polyhexametyleneguanidine

**Figure 9:**
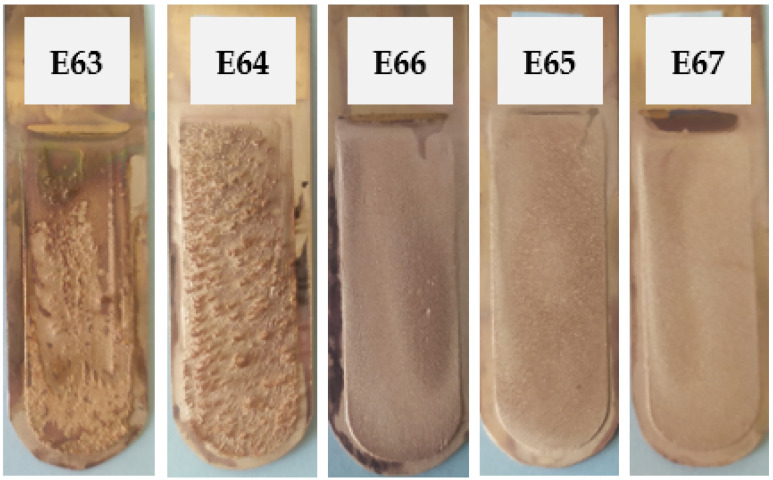
Appearance of the obtained cathode Cu deposits with the addition of ionic liquid based on polyhexamethylenebiguanidine (IL) and polyhexamethyleneguanidin (P) at concentrations of 0.005 mg/dm^3^ IL + 0.5 mg/dm^3^ P (E63), 0.05 mg/dm^3^ IL + 0.5 mg/dm^3^ P (E64), 0.15 mg/dm^3^ IL + 0.5 mg/dm^3^ P (E66), 0.5 mg/dm^3^ IL + 0.5 mg/dm^3^ P (E65), and 5.0 mg/dm^3^ IL + 0.5 mg/dm^3^ P (E67).

The last set of inhibitors introduced into the electrolyte was a set consisting of an ionic liquid based (IL) and polyhexamethyleneguanidin (P) at the concentrations outlined in Table 3. The appearance of the cathode Cu deposits is presented in Figure 9. Table 18 presents the average profile roughness parameters of the obtained electrolytic Cu deposit.

The cathode deposits obtained in tests E63 and E64 were characterized by a large number of growths, bubbles, and irregularities. In the case of the next three tests, E66, E65, and E67, the cathode copper was smoother, free of defects, and had a shiny crystalline structure. However, the roughness parameter values were not as low as those in the case of the E2 trial with the classic addition of inhibitors.

Table 19 presents the indicators the E44, E45, E60, and E61 electrorefining processes, as well as the evaluation of the cathode deposit quality.

The obtained current indicators were characterized by values higher than those in the case of the comparative test with the addition of bone glue and thiourea.

In summary, new sets of electrolyte additives in copper electrorefining help to control the morphology and quality of the cathode surface, but their levels need to be carefully controlled to avoid adverse effects. The additives absorb onto the cathode surface and participate in the electrochemical crystallization process. New sets of additives (particularly ionic liquid based on polyhexamethylenebiguanidine with bone glue, thiourea, and safranin) can be added to the acidic electrolyte to prevent nodulation and control the chemical and physical properties of copper cathodes. The new sets allow for the fragmentation of the crystalline structure of the copper cathode deposit and inhibit the growth and elimination of dendritic outgrowths (so-called dendrites) on the surface and edges of the cathodes.

## 4. Conclusions

The primary objective of the conducted research was to evaluate the effectiveness of new organic substances in copper electrorefining. These substances included an ionic liquid based on polyhexamethylenebiguanidine, polyhexamethyleneguanidine, safranin, and their combinations with other inhibitors such as bone glue and thiourea. The experiments were carried out on a small laboratory scale using industrial copper anodes sourced from local copper electrorefineries. The experiments were designed with the expectation of enhancing the current indicators and the smoothness of the obtained copper cathode deposits.

Single doses of inhibitors did not improve the process indicators, such as current efficiency, specific energy consumption, and average cell voltage. The surfaces of copper cathodes, depending on the initial concentration of the selected inhibitor, were characterized by minor defects, scratches, irregularities, or larger bubbles and dendrites. This was particularly noticeable in the E8 trial with the addition of safranin.

The introduction of a set, comprising a new ionic liquid based on polyhexamethylenebiguanidine and thiourea, led to a satisfactory current efficiency of approximately 97%, an average cell voltage of around 0.110 V, a low specific energy consumption index of roughly 100 kWh/t_Cu_, and smooth cathode copper surfaces with the lowest average profile roughness parameters. These values were inferior to those achieved in the E2 test with industrial inhibitors—bone glue and thiourea.

The results of these tests contribute to our understanding of how these organic substances and their combinations influence the electrorefining process. This knowledge can potentially lead to the development of more efficient and sustainable methods for copper electrorefining. Further research and testing are recommended to validate these findings and explore their practical applications in industrial settings.

## 5. Patents

In 2022, a patent application number P.442164 entitled “a method of producing high-purity electrolytic copper with using ionic liquid based on polyhexamethylenebiguanidine” was submitted to the Patent Office of the Republic of Poland.

## Figures and Tables

**Table 1:** Evaluation of the copper surface appearance.

Number of Points	Surface Appearance
0	Smooth deposit
1	Rough sediment, single surface irregularities
2	Distinct surface irregularities of copper, growths on the surface
3	Dendritic sediment, growths loosely associated with the cathode surface falling off the cathode or not covered with copper sediment

**Table 2:** Evaluation of the copper crystalline structure.

Number of Points	Crystalline Structure
0	Very fine, even crystal, matte surface
1	Small crystal, noticeable often on a fragment of the surface
2	Distinct, medium crystal, shiny surface
3	Coarse crystalline precipitate

**Table 3:** The initial concentrations of additives introduced into the electrolyte in the process of copper electrorefining—small laboratory scale.

Trial	Initial Concentration in Electrolyte, mg/dm^3^				
	IL	P	S	BG	T
E1	-	-	-	-	-
E2	-	-	-	5	5
E3	-	-	10	-	-
E4	-	-	25	-	-
E5	-	-	50	-	-
E6	-	0.05	-	-	-
E7	-	0.25	-	-	-
E8	-	0.50	-	-	-
E16	0.005	-	-	-	-
E9	0.01	-	-	-	-
E10	0.05	-	-	-	-
E12	0.50	-	-	-	-
E13	5	-	-	-	-
E14	50	-	-	-	-
E15	250	-	-	-	-
E19	0.005	-	-	5	-
E31	0.05	-	-	5	-
E46	0.15	-	-	5	-
E32	0.50	-	-	5	-
E34	5	-	-	5	-
E18	0.005	-	-	-	5
E22	0.05	-	-	-	5
E47	0.15	-	-	-	5
E24	0.50	-	-	-	5
E26	5	-	-	-	5
E44	0.005	-	50	-	-
E45	0.05	-	50	-	-
E60	0.15	-	50	-	-
E61	0.50	-	50	-	-
E63	0.005	0.50	-	-	-
E64	0.05	0.50	-	-	-
E66	0.15	0.50	-	-	-
E65	0.50	0.50	-	-	-
E67	5	0.50	-	-	-

**Table 4:** Average profile roughness parameters of the E1 and E2 copper deposits.

Trial	Average Profile Roughness Parameters, µm					
	Horizontal			Vertical		
	R_A_	R_Z_	R_m_	R_A_	R_Z_	R_m_
E1	5.26	29.30	33.62	4.78	28.03	31.92
E2	0.73	5.40	8.37	0.81	5.73	7.91

**Table 5:** The indicators for the E1 the E2 electrorefining processes with an evaluation of the Cu deposit quality.

Trial	*η*, %	*V_cell_*, *V*	SEC, kWh/t_Cu_	Evaluation of Cathode Deposit Quality		
				Surface Appearance	Crystalline Structure	Total Points
E1	100.0	0.180	147.4	1	2	3
E2	97.4	0.126	109.1	0	0	0

**Table 6:** Average profile roughness parameters of the E3, E4, and E5 copper deposits.

Trial	Average Profile Roughness Parameters, µm					
	Horizontal			Vertical		
	R_A_	R_Z_	R_m_	R_A_	R_Z_	R_m_
E3	2.92	17.00	19.18	4.54	25.15	33.37
E4	4.59	27.59	41.53	2.83	19.15	25.27
E5	1.61	10.97	14.29	1.64	11.35	15.54

**Table 7:** The indicators of the E3, E4, and E5 electrorefining processes with the evaluation of the Cu deposit quality.

Trial	*η*, %	*V_cell_*, *V*	SEC, kWh/t_Cu_	Evaluation of Cathode Deposit Quality		
				Surface Appearance	Crystalline Structure	Total Points
E3	99.9	0.128	107.0	2	1	3
E4	98.4	0.118	101.1	2	1	3
E5	98.2	0.101	86.7	1	0	1

**Table 8:** Average profile roughness parameters of the E6, E7, and E8 copper deposits.

Trial	Average Profile Roughness Parameters, µm					
	Horizontal			Vertical		
	R_A_	R_Z_	R_m_	R_A_	R_Z_	R_m_
E6	2.76	17.31	18.78	2.68	16.32	20.07
E7	-	-	-	8.56	44.42	51.05
E8	-	-	-	-	-	-

**Table 9:** The indicators of the E6, E7, and E8 electrorefining processes with the evaluation of the Cu deposit quality.

Trial	*η*, %	*V_cell_*, *V*	SEC, kWh/t_Cu_	Evaluation of Cathode Deposit Quality		
				Surface Appearance	Crystalline Structure	Total Points
E6	97.5	0.122	105.5	1	2	3
E7	99.2	0.156	132.6	1	2	3
E8	97.1	0.186	161.6	3	1	4

**Table 10:** Average profile roughness parameters of the E16, E9, E10, E12, E13, E14, and E15 copper deposits.

Trial	Average Profile Roughness Parameters, µm					
	Horizontal			Vertical		
	R_A_	R_Z_	R_m_	R_A_	R_Z_	R_m_
E16	9.34	48.74	59.04	10.26	51.72	59.67
E9	4.12	31.31	51.96	2.57	15.34	20.24
E10	2.75	15.93	19.44	2.96	18.29	22.42
E12	6.73	41.87	51.18	5.65	33.48	39.69
E13	10.75	53.74	62.30	8.95	44.85	59.86
E14	6.54	35.67	46.56	7.61	39.80	49.77
E15	8.96	56.60	64.75	7.68	42.34	52.15

**Table 11:** The indicators of the E16, E9, E10, E12, E13, E14, and E15 electrorefining processes with the evaluation of the Cu deposit quality.

Trial	*η*, %	*V_cell_*, *V*	SEC, kWh/t_Cu_	Evaluation of Cathode Deposit Quality		
				Surface Appearance	Crystalline Structure	Total Points
E16	97.5	0.138	119.3	0	2	2
E9	99.2	0.157	133.5	1	0	1
E10	98.4	0.165	141.4	1	0	1
E12	97.7	0.142	122.6	0	1	1
E13	99.1	0.153	130.2	2	2	4
E14	96.9	0.171	148.8	2	2	4
E15	94.1	0.238	213.3	0	1	1

**Table 12:** Average profile roughness parameters of the E19, E31, E46, E32, and E34 copper deposits.

Trial	Average Profile Roughness Parameters, µm					
	Horizontal			Vertical		
	R_A_	R_Z_	R_m_	R_A_	R_Z_	R_m_
E19	8.06	47.26	56.99	7.11	40.68	54.27
E31	2.68	16.50	19.38	2.60	16.62	18.64
E46	3.75	22.61	24.89	3.55	21.09	24.06
E32	3.02	17.91	20.52	2.97	17.67	20.63
E34	3.81	22.46	25.91	3.93	22.95	26.61

**Table 13:** The indicators of the E19, E31, E46, E32, and E34 electrorefining processes with the evaluation of the Cu deposit quality.

Trial	*η*, %	*V_cell_*, *V*	SEC, kWh/t_Cu_	Evaluation of Cathode Deposit Quality		
				Surface Appearance	Crystalline Structure	Total Points
E19	99.6	0.158	133.8	0	2	2
E31	98.3	0.173	148.6	0	0	0
E46	98.5	0.179	153.3	0	1	1
E32	98.2	0.163	140.0	0	0	0
E34	99.9	0.199	167.5	0	1	1

**Table 14:** Average profile roughness parameters of the E18, E22, E47, E24, and E26 copper deposits.

Trial	Average Profile Roughness Parameters, µm					
	Horizontal			Vertical		
	R_A_	R_Z_	R_m_	R_A_	R_Z_	R_m_
E18	1.26	8.35	10.87	1.18	8.02	9.80
E22	1.12	7.05	7.86	1.27	8.11	10.60
E47	1.02	7.11	8.54	1.05	6.62	8.00
E24	0.91	5.87	7.23	1.00	6.99	8.96
E26	1.70	11.12	46.96	1.63	10.73	13.79

**Table 15:** The indicators of the E18, E22, E47, E24, and E26 electrorefining processes with the evaluation of the cathode deposit quality.

Trial	*η*, %	*V_cell_*, *V*	SEC, kWh/t_Cu_	Evaluation of Cathode Deposit Quality		
				Surface Appearance	Crystalline Structure	Total Points
E18	97.1	0.113	98.0	0	0	0
E22	96.9	0.116	101.0	0	0	0
E47	96.1	0.113	99.2	0	0	0
E24	97.4	0.118	102.2	0	0	0
E26	97.7	0.174	150.3	0	0	0

**Table 16:** Average profile roughness parameters of the E44, E45, E60, and E61 copper deposits.

Trial	Average Profile Roughness Parameters, µm					
	Horizontal			Vertical		
	R_A_	R_Z_	R_m_	R_A_	R_Z_	R_m_
E44	2.16	13.00	15.41	2.34	15.09	17.73
E45	4.86	27.13	28.56	4.86	27.69	31.32
E60	3.64	21.10	24.68	3.64	21.60	28.32
E61	3.94	26.50	30.15	4.06	26.13	32.39

**Table 17:** The indicators of the E44, E45, E60, and E61 processes with the evaluation of the Cu deposit quality.

Trial	*η*, %	*V_cell_*, *V*	SEC, kWh/t_Cu_	Evaluation of Cathode Deposit Quality		
				Surface Appearance	Crystalline Structure	Total Points
E44	97.9	0.108	93.1	0	1	1
E45	96.7	0.116	101.2	0	1	1
E60	100.0	0.149	125.6	0	1	1
E61	98.5	0.201	172.1	0	1	1

**Table 18:** Average profile roughness parameters of the E63, E64, E66, E65, and E67 copper deposits.

Trial	Average Profile Roughness Parameters, µm					
	Horizontal			Vertical		
	R_A_	R_Z_	R_m_	R_A_	R_Z_	R_m_
E63	-	-	-	-	-	-
E64	-	-	-	-	-	-
E66	4.82	30.33	44.59	4.73	26.67	30.65
E65	5.51	36.41	45.88	5.55	32.26	40.92
E67	4.90	26.47	31.16	4.53	26.48	30.81

**Table 19:** The indicators of the E63, E64, E66, E65, and E67 electrorefining processes with the evaluation of the cathode deposit quality.

Trial	*η*, %	*V_cell_*, *V*	SEC, kWh/t_Cu_	Evaluation of Cathode Deposit Quality		
				Surface Appearance	Crystalline Structure	Total Points
E63	98.5	0.230	197.0	3	3	6
E64	99.1	0.215	183.1	3	3	6
E66	99.2	0.147	124.9	1	2	3
E65	98.1	0.159	137.0	1	2	3
E67	98.5	0.297	254.3	1	2	3

## Data Availability

Data are contained within the article.
